# Genome-wide association analysis of panicle exsertion and uppermost internode in rice (*Oryza sativa* L.)

**DOI:** 10.1186/s12284-019-0330-x

**Published:** 2019-09-18

**Authors:** Chengfang Zhan, Jiaxiao Hu, Qiao Pang, Bin Yang, Yanhao Cheng, Enshun Xu, Peiwen Zhu, Yingyi Li, Hongsheng Zhang, Jinping Cheng

**Affiliations:** Laboratory of Seed Science and Technology, State Key Laboratory of Crop Genetics and Germplasm Enhancement, Jiangsu Collaborative Innovation Center for Modern Crop Production, Nanjing Agricultural University, Nanjing, China

**Keywords:** *Oryza sativa* L., Genome-wide association study, Panicle exsertion (PE), Uppermost internode (UI), Panicle enclosure

## Abstract

**Background:**

Rice (*Oryza sativa* L.) yield is seriously influenced by panicle exsertion (PE) and the uppermost internode (UI) through panicle enclosure or energy transport during grain-filling stages. We evaluated the traits of PE and UI of 205 rice accessions in two independent environments and performed genome-wide association (GWAS) to explore the key genes controlling PE and UI, which could be used to improve panicle enclosure in rice breeding.

**Results:**

In this study, extensive genetic variation was found in both PE and UI among the 205 rice accessions, and 10.7% of accessions had panicle enclosure (PE/UI ≤ 0). Correlation analysis revealed that PE was significantly positively correlated with 1000-grain weight (1000-GW) but negatively correlated with heading date (HD), and UI was significantly positively correlated with HD but no significantly correlated with 1000-GW. A total of 22 and 24 quantitative trait loci (QTLs) were identified for PE and UI using GWAS, respectively. Eight loci for PE and nine loci for UI were simultaneously detected both in 2015 and in 2016, seven loci had adjacent physical positions between PE and UI, and ten loci for PE and seven loci for UI were located in previously reported QTLs. Further, we identified the *CYP734A4* gene, encoding a cytochrome P450 monooxygenase, and the *OsLIS-L1* gene, encoding a lissencephaly type-1-like protein, as causal genes for *qPE14* and *qUI14*, and for *qPE19*, respectively. PE and UI were both significantly shorter in these two genes’ mutants than in WT. Allelic Hap.1/2/4 of *CYP734A4* and Hap.1/2/4 of *OsLIS-L1* increased PE, UI, PE/UI, and 1000-GW, but Hap.3 of *CYP734A4* and Hap.3 of *OsLIS-L1* reduced them. In addition, six candidate genes were also detected for four key novel loci, *qPE16*, *qPE21*, *qUI1*, and *qUI18*, that seemed to be related to PE and UI.

**Conclusions:**

Our results provide new information on the genetic architecture of PE and UI in rice, confirming that the *CYP734A4* and *OsLIS-L1* genes participate in PE and UI regulation, which could improve our understanding of the regulatory mechanism of PE and UI for rice breeding in the future.

## Background

Rice is a staple food for nearly half of the world’s population. Rice yield is seriously reduced when panicle enclosure occurs in cultivated varieties, particularly in hybrid rice varieties (Guan et al. 2011; Duan et al. 2012). It is well known that panicle exsertion (PE) and the uppermost internode (UI) play a critical role in the regulation of panicle enclosure (da Cruz et al. 2008; Qiao et al. 2008; Duan et al. 2012). Panicle enclosure means that panicles are partly or fully enclosed within the flag leaf sheath, mainly caused by the shortening of UI (Yin et al. 2007; Guan et al. 2011). Simultaneously, both PE and UI are connected to the culm and panicle, controlling the transport efficiency of water and nutrients from the leaves and stems to grains and ultimately affecting grain filling and crop yield (Ma et al. 2002; da Cruz et al. 2008; Liu et al. 2008). Therefore, exploring the key genes related to PE and UI in rice is helpful for breeding high-yield rice varieties without panicle enclosure by the marker-assisted selection (MAS) approach.

PE and UI have been reported to be quantitative traits controlled by multiple genes, and some quantitative trait loci (QTLs) have been identified by the traditional mapping approach in rice (Qiao et al. 2008; Zhao et al. 2016; Herlina et al. 2016). Qiao et al. (2008) identified three QTLs for UI on chromosomes 1, 3 and 6 and four QTLs for panicle enclosure on chromosomes 1, 3, 5, and 10, respectively. Zhao et al. (2016) detected five QTLs, *qPE1*, *qPE3*, *qPE6*, *qPE9*, and *qPE11*, for PE. One QTL for incomplete panicle exertion was identified on chromosome 4 using a BC_2_F_8_ line derived from a cross between IR64 and Gajah Mungkur (Herlina et al. 2016). Recently, a *qPE12* locus controlling rice panicle exsertion was fine-mapped using C115, a chromosome segment substitution line carrying introgression segments of Nipponbare on the genetic background of *indica* variety 9311 (Zhao et al. 2018).

So far, at least 6 mutants of PE and UI have been reported in rice, and their corresponding genes were mapped or cloned, such as *ESP2* (Guan et al. 2011) and *sui1/OsPSS-1* for UI on chromosome 1 (Zhu et al. 2011), *HOX12* for PE and UI on chromosome 3 (Gao et al. 2016), *EUI1* for UI on chromosome 5 (Lou et al. 2006), *SUI4* for UI on chromosome 7 (Ji et al. 2014), and *ESP1* for UI on chromosome 11 (Duan et al. 2012). Of these genes, the shortened uppermost internode 1 (*SUI1*) gene was belonged to the SUI family, which encodes base-exchange types of phosphatidyl serine synthases (PSS) (Yin et al. 2013). And, the *SUI1* gene was found to negatively regulate rice UI by secreting cell wall components (Zhu et al. 2011; Ma et al. 2016). *EUI1* was obtained by map-based cloning and corresponded to a P450 gene, *CYP714D1*, encoding a cytochrome P450 monooxygenase, and elongated UI by causing accumulation of gibberellin (GA) (Ma et al. 2006; Lou et al. 2006; Zhu et al. 2006). Gao et al. (2016) found that the homeodomain-leucine zipper transcription factor HOX12 acted directly through *EUI1* to regulate PE. In addition, the *OsLIS-L1* gene (*lissencephaly type-1-like 1*) encodes the lissencephaly type-1-like protein containing the WD40 motif, which was reported to play an important role in rice UI (Gao et al. 2012).

With the rapid development of high-throughput sequencing technology, genome-wide association studies (GWAS) are becoming an effective approach to identify genes underlying complex traits in rice. Huang et al. (2010) performed a GWAS to analyse 14 agronomic traits, including grain width, grain weight and heading date, via 517 sequenced rice landraces. Famoso et al. (2011) identified aluminium tolerance by a GWAS based on 383 rice accessions. Jia et al. (2012) reported that ten loci on seven chromosomes were significantly associated with the response to the sheath blight pathogen using 217 rice accessions. Many panels of high-density single-nucleotide polymorphism (SNP) data have been built to explore new genes related to target traits by GWAS (Han and Huang, 2013; Li et al. 2014). Through 413 different accessions of *O. sativa* collected from 82 countries, high-resolution genotyping panels including 44,100 and 700,000 SNP variants were built for GWAS by Zhao et al. (2011) and McCouch et al. (2016), respectively. The 3000 Rice Genomes Project was carried out based on the sequencing of 3000 rice accessions that afforded a global representation of genetic and functional diversity (Li et al. 2014). Furthermore, the estimated effect of nucleotide polymorphisms was created to rapidly identify candidate genes with traits from GWAS (Yano et al. 2016). There are no reports about genes associated with PE and UI that have been identified via GWAS in rice.

In this study, PE and UI traits on a targeted population of 205 rice accessions (114 *INDICA* accessions, 57 *JAPONICA* accessions, and 34 *ADMIX* accessions) from rice diversity pane l (RDP1) (Zhao et al. 2011) were investigated to detect the associated loci via GWAS using a high-resolution, open-access research platform that included 700 K SNP data (McCouch et al. 2016). Heading date (HD) and 1000-grain weight (1000-GW) were also analysed to examine the relationships among PE, UI, HD and 1000-GW. The functional SNPs with each main peak-associated locus were estimated to refine the target candidate genes for further research. Our studies indicated that *CYP734A4* and *OsLIS-L1* genes could participate in PE and UI regulation, and six genes within four key novel loci seemed to be related to PE and UI.

## Results

### Phenotypic variance

**Fig. 1:**
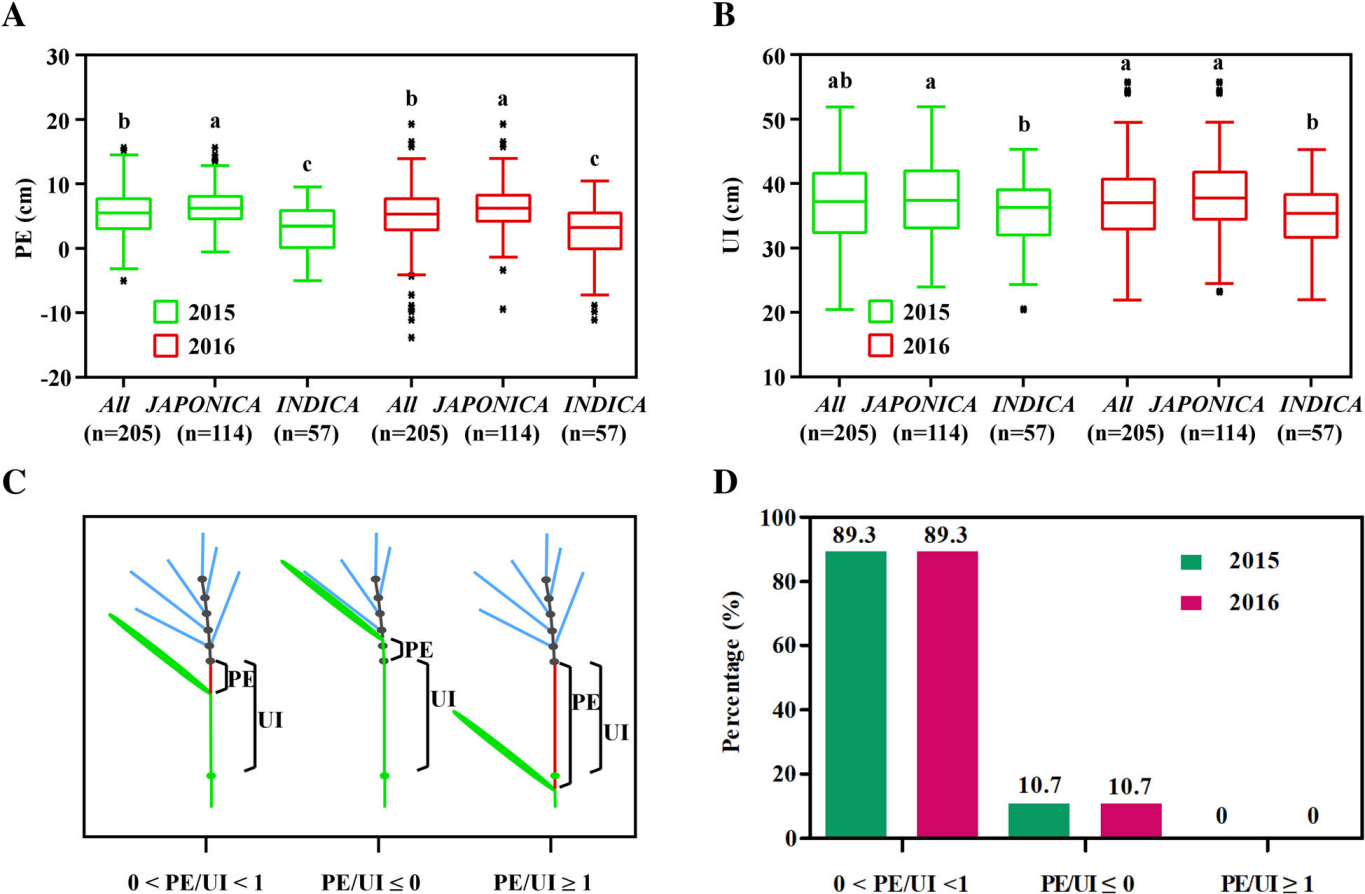
Phenotypic distribution in the RDP1 in 2015 and 2016. **a**, **b** Box plot of the phenotypic variation of PE and UI within the *All*, *JAPONICA* and *INDICA* groups. The horizontal lines represent the median values, box edges represent upper and lower quantiles, and whiskers are 1.5 times the quantile of the data. Outliers are shown as open dots. n represents the number of genotypes, and small letters above error bars indicate significant differences between subpopulations (LSD, *P* < 0.05). **c** Three pattern diagrams between PE and UI. 0 < PE/UI < 1 represents flag leaf overlapping a part of PE, PE/UI ≤ 0 represents flag leaf enclosing the panicle partly or fully, and PE/UI ≤ 0 represents flag leaf under the node of UI. **d** Analysis of frequency distribution for the three models between PE and UI

The PE and UI traits of 205 rice accessions from RDP1 (Zhao et al. 2011) were evaluated in 2015 and 2016 in the fields at Nanjing, China (Fig. 1a, b; Additional file 5: Table S2). Phenotype analysis showed that 89.3% of accessions had their flag leaves overlapping a part of PE (0 < PE/UI < 1), 10.7% had panicle enclosure (PE/UI ≤ 0), and none had its flag leaf under the node of UI (PE/UI ≥ 1) (Fig. 1c, d). The results revealed that this population had a large phenotypic variation in PE and UI traits, ranging from − 13.92 to 19.24 cm and 20.53 to 56.01 cm, respectively (Additional file 5: Table S1).

The coefficient of variation (CV) for PE ranged from 51.30 to 116.78% and 60.31 to 211.40%, with mean values of 3.00 to 6.34 cm and 2.30 to 6.38 cm in 2015 and 2016, respectively. The broad-sense heritability of PE was estimated to be 33.89 to 80.93%. The CV for UI ranged from 15.48 to 17.04% and 15.09 to 16.31%, with mean values of 35.31 to 37.75 cm and 34.83 to 37.95 cm in 2015 and 2016, respectively. The broad-sense heritability of UI was estimated to be 47.32 to 96.19% (Additional file 5: Table S1). 1000-GW and HD were also evaluated, and their broad-sense heritability was estimated as 81.32 to 85.14% and 79.82 to 84.90%, respectively (Additional file 5: Table S1). By comparison, the mean values of PE and UI in the *INDICA* group were both significantly lower than those in the *JAPONICA* group (Fig. 1a, b). These results indicated that the population had extensive genetic variation in PE and UI traits.

### Correlation analysis

The correlation analysis was performed for twelve pairwise combinations derived from PE, UI, HD, and 1000-GW traits (Additional file 1: Figure S1). The results showed that there were similar correlations between them in 2015 and 2016. An extremely significantly positive correlation was found between PE and UI. Meanwhile, the correlation analysis showed that PE had a significantly positive correlation with 1000-GW and a significantly negative correlation with HD. UI had a significantly positive correlation with HD but no significant correlation with GW. In addition, there was a significantly negative correlation between 1000-GW and HD.

### GWAS of PE and UI

**Fig. 2:**
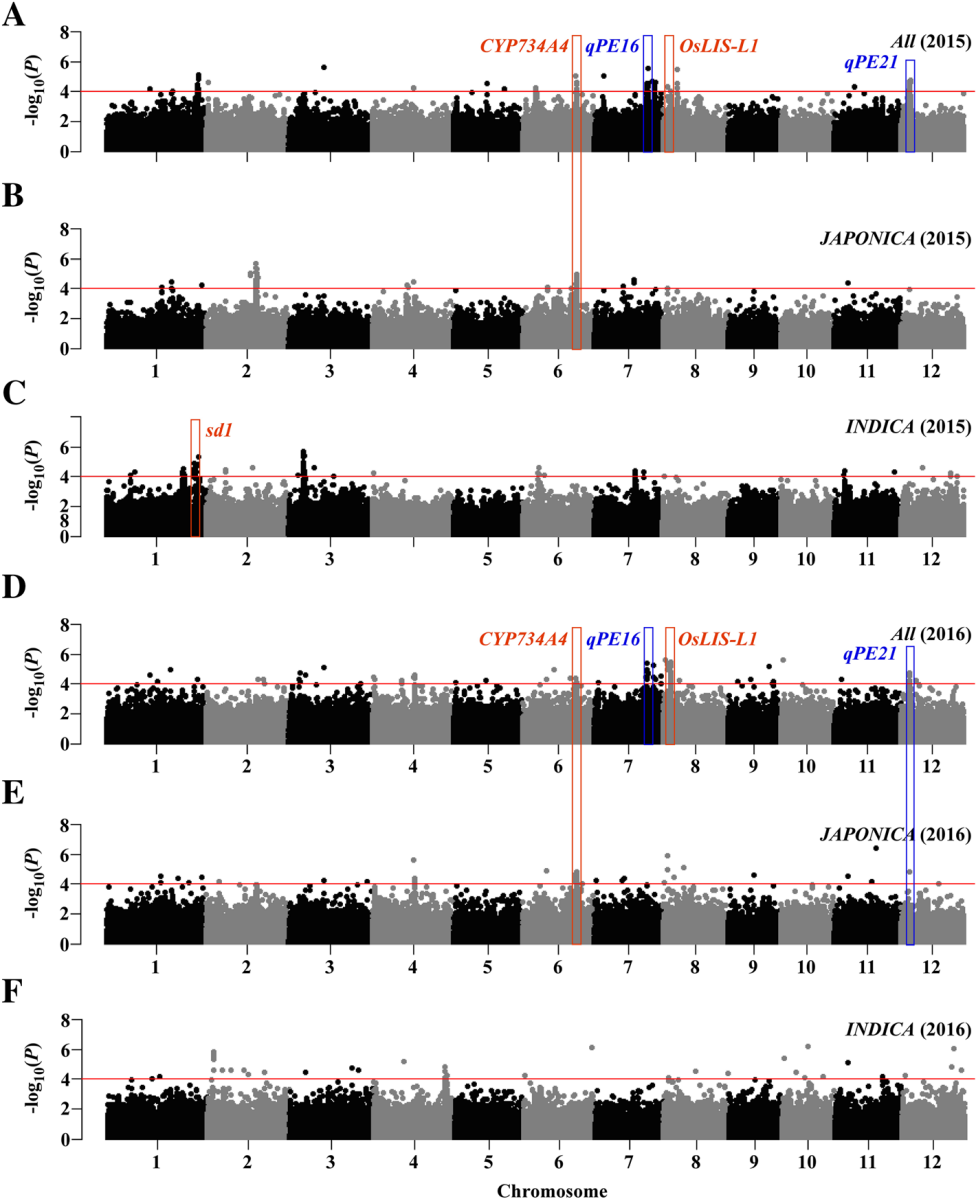
Genome-wide Manhattan plots of association mapping for PE using the EMMAX model. Associations identified based on *All* (2015, **a**; 2016, **d**), *JAPONICA* (2015, **b**; 2016, **e**) and *INDICA* (2015, **c**; 2016, **f**) groups are depicted in separate panels. The red bar frames indicate the genes reported previously, and the blue bar frames indicate the key novel loci co-located in both 2015 and 2016. The x axis depicts the physical location of SNPs across the 12 chromosomes of rice, and the y axis depicts -log_10_(*P* value). Significant SNPs with *P* ≤ 0.0001 are denoted as red lines

**Fig. 3:**
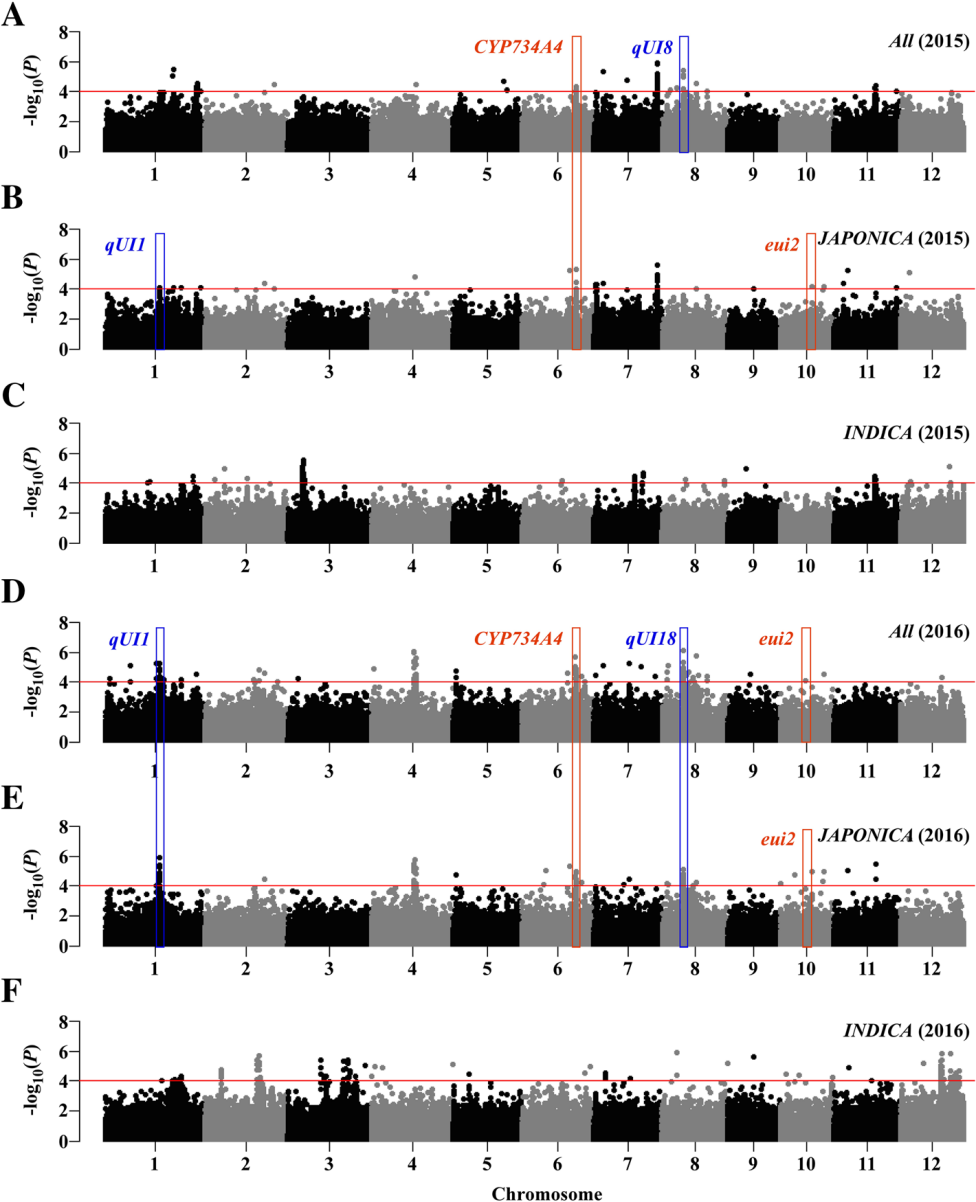
Genome-wide Manhattan plots of association mapping for UI using the EMMAX model. Associations identified based on *All* (2015, **a**; 2016, **d**), *JAPONICA* (2015, **b**; 2016, **e**) and *INDICA* (2015, **c**; 2016, **f**) groups are depicted in separate panels. The red bar frames indicate that genes are reported, and the blue bar frames indicate the key novel loci co-located in both 2015 and 2016. The x axis depicts the physical location of SNPs across 12 chromosomes of rice, and the y axis depicts -log_10_(*P* value). Significant SNPs with *P* ≤ 0.0001 are denoted as red lines

**Fig. 4:**
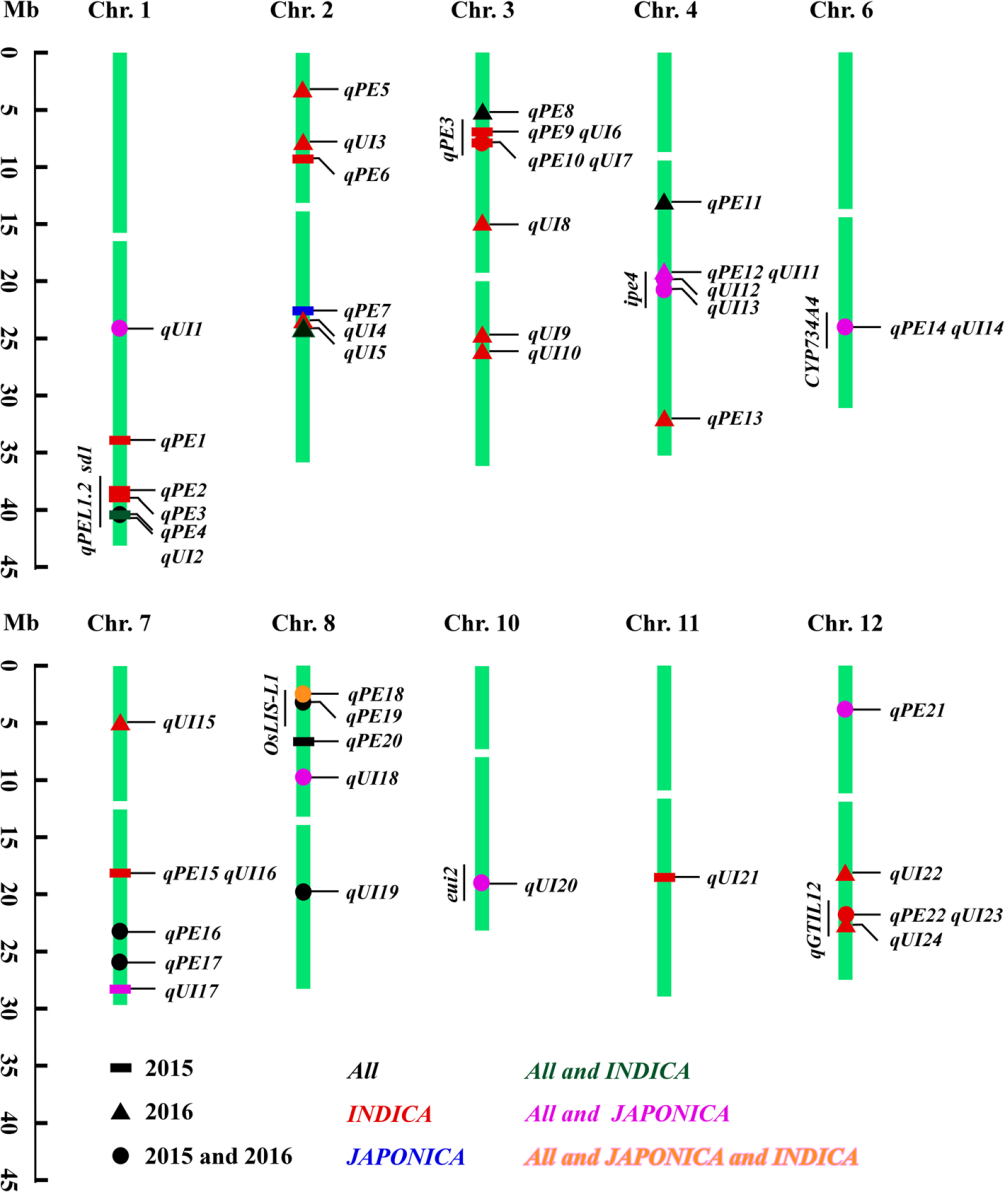
Chromosomal positions of loci for PE and UI identified via GWAS in *All*, *JAPONICA*, and *INDICA* groups in 2015 and 2016. The mapped loci in this study are shown on the right, and previously mapped QTLs are shown on the left

We carried out GWAS on the whole population as well on individual subpopulations based on large variation within the *JAPONICA* and *INDICA* subpopulations (Fig. 1a, b). The analysis showed that 22 loci within 261 SNPs on eight rice chromosomes were significantly associated with PE, and 24 loci within 278 SNPs on 10 chromosomes were significantly associated with UI (Figs. 2 and 3; Additional file 5: Table S3). Among those loci, eight PE-related and nine UI-related loci were co-located in both 2015 and 2016, and seven pairs of loci had adjacent physical positions between PE and UI (Figs. 2, 3 and 4; Additional file 5: Table S3). In our analysis of subpopulations, five loci for PE and eight loci for UI were detected in *JAPONICA*, and 12 loci for PE and 14 loci for UI were found in *INDICA*. Among them, five loci for PE and 11 loci for UI were found in both *All* and sub-populations (Figs. 2, 3 and 4; Additional file 5: Table S3).

By comparison, ten loci for PE and seven loci for UI were simultaneously identified with the reported mapped loci or characterized genes (Fig. 4; Additional file 5: Table S3). The remaining 12 loci for PE and 17 for UI might be novel. Of these novel loci, *qPE16*, *qPE21*, *qUI1* and *qUI18*, were co-located in both 2015 and 2016 with significantly leading SNP (*P* < 1.0e-8) (Figs. 2 and 3), and were considered to be further analyzed for causal genes in our study.

### Effect of *CYP734A4* and *OsLIS-L1* on PE and UI

**Fig. 5:**
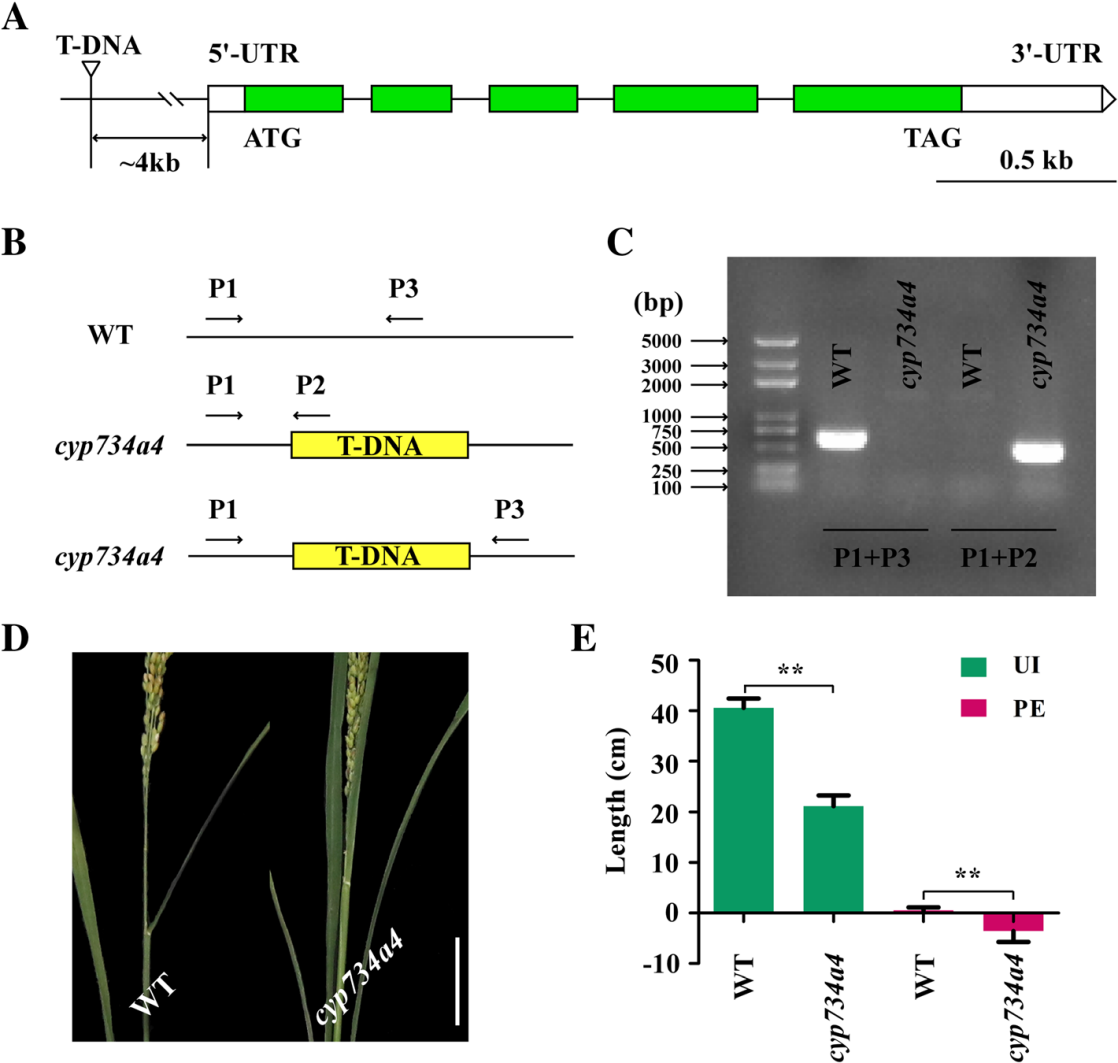
Identification of *CYP734A4* as the causal gene for *qPE14* and *qUI14.* **a** Schematic presentation of the gene structure of *CYP734A4* and T-DNA insertion sites in *cyp734a4* mutants. **b** P1/P2 and P1/P3 are two pairs of primers used to amplify *CYP734A4* DNA for PCR analysis. **c** PCR analysis of WT and *cyp734a4* mutants. **d**, **e** PE and UI of WT and *cyp734a4* mutants. ** indicates a significant difference compared to WT at the 1% level

**Fig. 6:**
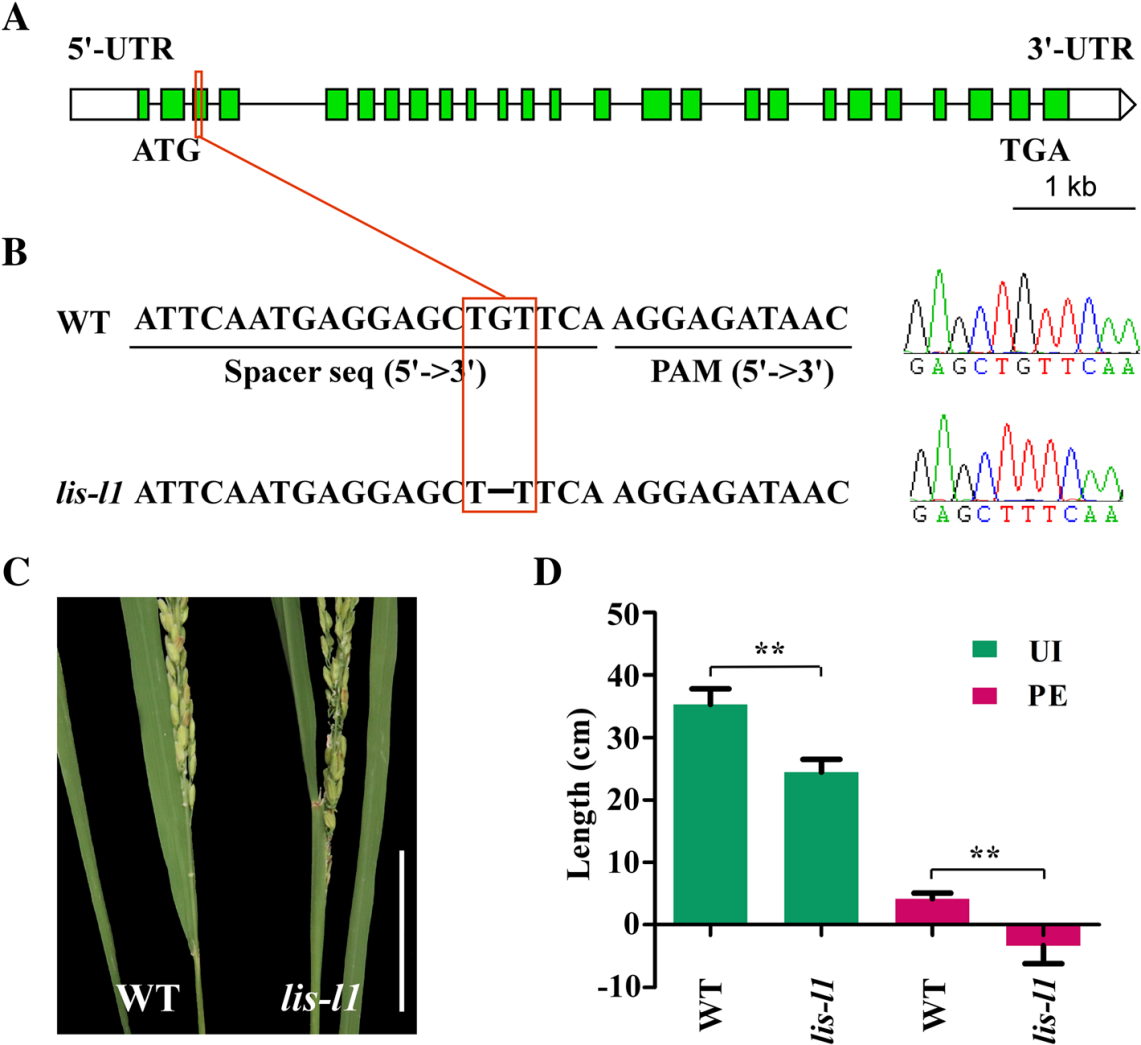
Identification of *OsLIS-L1* as the causal gene for *qPE19.* **a** Schematic presentation of the gene structure of *OsLIS-L1* and **b** deletion sites in *oslis-l1* mutants using the CRISPR/Cas9 system. **c**, **d** PE and UI of WT and *oslis-l1* mutants. ** indicates a significant difference compared to WT at the 1% level

According to previous reports, all internodes of *cyp734a4* mutants are significantly shortened by degrading brassinosteroids (BRs) (Qian et al. 2017), and the *OsLIS-L1* gene can regulate cell proliferation in the first internodes under the panicle (Gao et al. 2012). In our study, we found that the *CYP734A4* gene was located within *qPE14* and *qUI14*, and *OsLIS-L1* was located within *qPE19* (Figs. 2, 3 and 4). Therefore, we obtained homozygous mutants of *cyp734a4* containing a T-DNA insertion in the promoter region of the *CYP734A4* gene in Zhonghua11 (Fig. 5a-c) and structured the *oslis-l1* mutants by using the CRISPR/Cas9 system from the Nipponbare variety in this study (Fig. 6a, b). The results showed that PE and UI in *cyp734a4* mutants were significantly shorter than those in WT (Fig. 5d, e), with significant panicle enclosure. In *oslis-l1* mutants, PE and UI were also significantly reduced (Fig. 6d, e), which was similar to the results from Gao et al. (2012). These results indicate that the *CYP734A4* and *OsLIS-L1* genes might be target candidate genes for the *qPE14* and *qUI14*, and *qPE19* loci respectively.

### Haplotype analysis of *CYP734A4* and *OsLIS-L1*

**Fig. 7:**
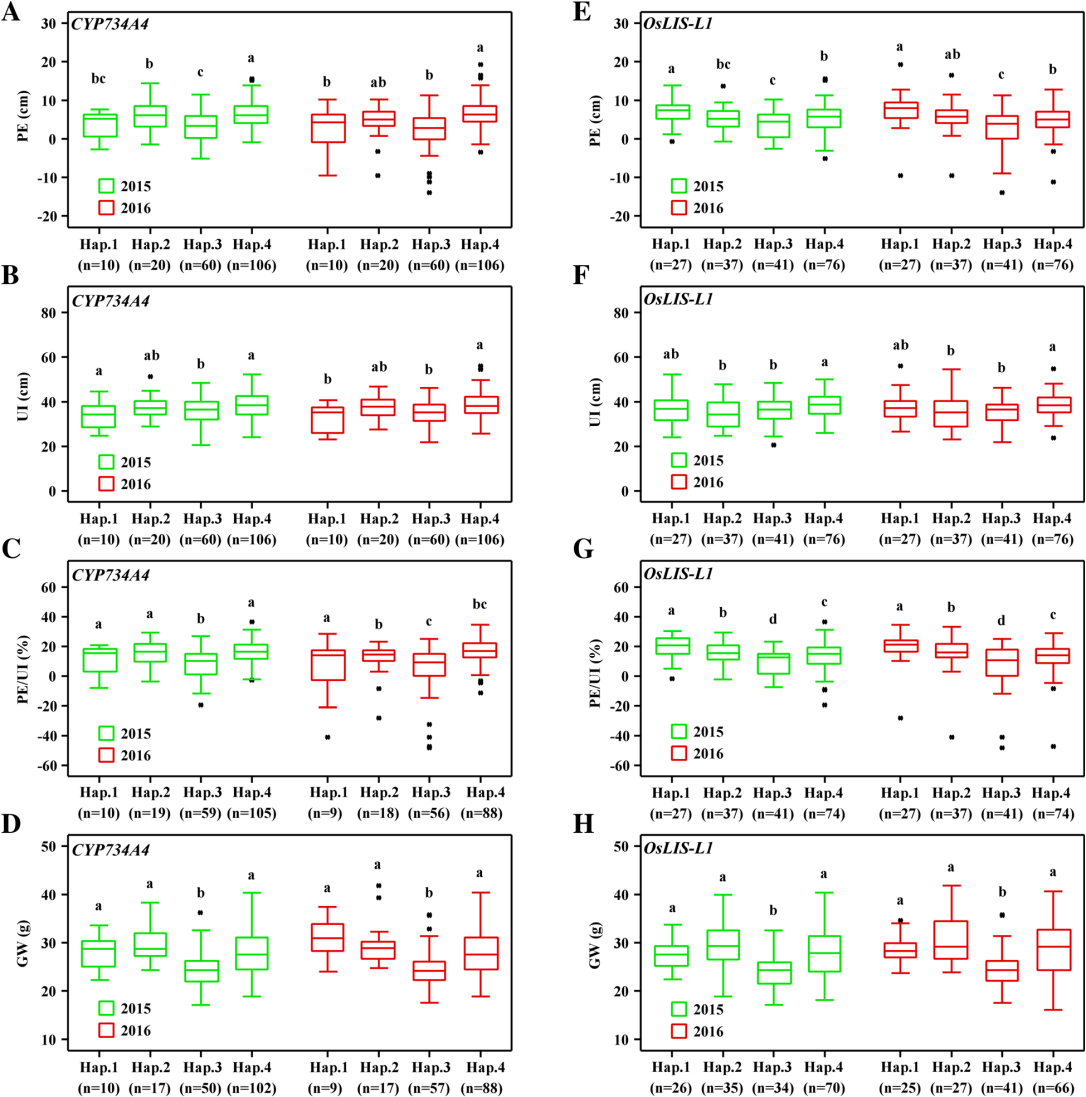
Box plots of PE, UI, PE/UI, and 1000-GW based on haplotypes of *CYP734A4* (**a-d**) and *OsLIS-L1* (**e-h**) in 2015 and 2016. n represents the number of accessions with a specific haplotype. The horizontal lines represent the median values, box edges represent upper and lower quantiles, and whiskers are 1.5 times the quantile of the data. Outliers are shown as open dots. Small letters above error bars indicate significant differences among haplotypes (LSD, *P* < 0.05). *CYP734A4*: Hap.1, AA; Hap.2, CN; Hap.3, AT; Hap.4, CA. *OsLIS-L1*: Hap.1, CCCAT; Hap.2, CTTAT; Hap.3, TCTGC; Hap.4, CCTAT

Based on all SNPs with an MAF > 0.05 within functional range of the 5′ flanking sequences of genes (≤2 kb from the first ATG), 4 and 4 major haplotypes in *CYP734A4* and *OsLIS-L1* were detected among the 205 accessions, respectively (Fig. 7). For the *CYP734A4* gene, the PE of Hap.3 was significantly lower than those of Hap.2 and Hap.4 in 2015 and Hap.4 in 2016 (Fig. 7a). The UI of Hap.3 was significantly lower than those of Hap.1 and Hap.4 in 2015 and Hap.4 in 2016 (Fig. 7b). The PE/UI of Hap.3 was significantly lower than those of Hap.1, Hap.2, and Hap.4 in 2015 and those of Hap.1 and Hap.2 in 2016 (Fig. 7c). For the O*sLIS-L1* gene, the PE of Hap.3 was significantly lower than those of Hap.1 and Hap.4 in 2015 and those of Hap.1, Hap.2, and Hap.4 in 2016 (Fig. 7e). The UI of Hap.3 was significantly lower than that of Hap.4 in both 2015 and 2016 (Fig. 7f). The PE/UI of Hap.3 was significantly lower than those of Hap.1, Hap.2, and Hap.4 in both 2015 and 2016 (Fig. 7g). The GW of Hap.3 for *CYP734A4* and Hap.3 for *OsLIS-L1* was significantly lower than those of Hap.1, Hap.2, and Hap.4 in both 2015 and 2016 respectively (Fig. 7d, h). These results suggest that Hap.3 of *CYP734A4* and Hap.3 of *OsLIS-L1* genes might result in lower values of PE, UI, PE/UI and 1000-GW via allelic variation within the promoter region (Additional file 2: Figure S2).

### Candidate genes involved in four novel QTLs

To identify candidate genes within the two novel key PE-related (*qPE16* and *qPE21*) and two UI-related QTLs (*qUI1* and *qUI18*), we analysed a 200 kb genomic region by comparing the related regions with the Nipponbare reference genome (http://rice.plantbiology.msu.edu) by GWAS based on the results in 2015 and 2016 (Figs. 2 and 3). A total of 306 candidate genes were identified among the 4 association peaks in the surrounding genomic areas after removal of genes encoding unknown/trotransposon/retrotransposon proteins (Additional file 5: Table S4).

Gene Ontology analysis showed that there was significant enrichment in 11 functional categories (http://bioinfo.cau.edu.cn/agriGO/analysis.php) (LSD < 0.05), including 6 functional categories under biological process, 4 functional categories under cellular component, and 1 functional category under molecular function (Additional file 3: Figure S3). Among them, membrane functional type (GO: 0016020) possessed more significant differences compared to other categories(Additional file 3: Figure S3; Additional file 5: Table S5).

Further, all the significant SNPs in the candidate regions were classified into five functional groups according to Yano et al. (2016), and 28 candidate genes were screened out, including 11 genes for *qPE16* on Chr. 7, six genes for *qPE21* on Chr. 12, seven genes for *qUI1* on Chr. 1, and four genes for *qUI8* on Chr. 8 (Additional file 5: Table S5).

By comparative analysis, we found that six genes, *LOC_Os01g41420* within *qUI1*, *LOC_Os07g38530* and *LOC_Os07g38810* within *qPE16*, *LOC_Os08g15170* and *LOC_Os08g15280* within *qUI18*, and *LOC_Os12g07690* within *qPE21*, belonged to the membrane functional type in Gene Ontology analysis. Of these, one significant SNP was located in the coding sequence (CDS) of a gene, and five were located in promoter regions (Additional file 5: Table S5).

## Discussion

In this study, none of the accessions with flag leaves under the node of the UI were found among 205 accessions from RDP1 (Zhao et al. 2011). Most of the accessions showed that the flag leaf overlapped a part of the UI, and only 10.7% of the accessions displayed panicle enclosure that would lead to reduced yield because of spikelet fertility. Statistical analysis revealed that RDP1 captures extensive natural variation in all accessions, possibly increasing the chance to identify new loci of PE and UI. Simultaneously, the extensive variation of both PE and UI were also found in either *JAPONICA* or *INDICA* groups in this study, which demonstrated that the two sub-populations could be used for GWAS. By comparison, we found that *JAPONICA* had significantly higher mean values than *INDICA* in both PE and UI, means that *japonica* varieties have longer PE and UI than *indica* varieties. Furthermore, most *INDICA* accessions with negative PE are from African countries (Additional file 5: Table S2), suggesting that *INDICA* rice may undergo panicle enclosure more easily, as reported by Guan et al. (2011).

As major components in the process of energy transportation, PE and UI have an important regulatory effect on grain filling and grain size (Zhao et al. 2016). In our study, we found that PE had a significantly positive correlation with 1000-GW, but UI had no significant correlation with 1000-GW. These results suggest that PE may be more important in the regulation of grain weight than UI. It is well known that HD is one of the most important agronomic traits in rice (Takahashi et al. 2009; Matsubara et al. 2011). Correlation analysis showed that HD had a significantly negative correlation with PE, which was similar to the results reported by Zhao et al. (2016), and a significantly positive correlation with UI. This suggests that HD has different impacts on PE and UI in rice in the field. Meanwhile, there was a significantly positive correlation between PE and UI, indicating that panicle enclosure can be prevented when extending UI in rice breeding.

To dig new QTLs controlling PE and UI would contribute to understanding its the genetic control and elucidating the phenomenon of panicle enclosure. In our study, we identified 46 associated loci based on 205 natural rice accessions from RDP1 via GWAS, including 22 loci from PE and 24 loci from UI. By comparing the chromosomal positions, 10 loci for PE and seven loci for UI in our study were located in the previously reported QTLs/genes. The *qPE2* was near the region of *qPE1* (Zhao et al. 2016), and *qPE3* co-located with *sd1* (Ayano et al. 2014). The *qPE8*, *qPE9*, *qPE10*, *qUI6*, and *qUI7* locations were all close to the region of *qPE3* (Zhao et al. 2016). *qPE22*, *qUI23*, and *qUI24* were all located near the region of *qGTIL12* (Nagai et al. 2014). *qPE13* was near the region of *ipe.4.1* (Herlina et al. 2016). *qUI2* and *qUI20* were located within *qPEL1.2* and *eui2*, respectively (Dang et al. 2017). *qPE19* was co-located with *OsLIS-L1* (Gao et al. 2012). *qPE14* and *qUI14* were co-located with *CYP734A4* (Qian et al. 2017). However, there were still 12 loci for PE and 17 loci for UI that had not been previously reported, which might be novel QTLs.

By comparing these QTLs, we found that more loci in *INDICA* were identified than in *JAPONICA*, and 15 loci for PE and 22 loci for UI were not shared between in *INDICA* and *JAPONICA*, supporting the earlier notion that *JAPONICA* and *INDICA* were domesticated independently from different geographical and ecological locations (Kovach et al. 2007). Eight PE-related and nine UI-related loci were showed to be simultaneously detected in both 2015 and 2016 as inter-year comparison, suggestting that they are relatively stable and less affected by the environment. Besides, we found that seven pairs of loci, including *qPE4* and *qUI2* on chromosome 1, *qPE9* and q*UI6* on chromosome 3, *qPE10* and *qUI7* on chromosome 3, *qPE12* and *qUI11* on chromosome 4, *qPE14* and *qUI14* on chromosome 6, *qPE15* and *qUI16* on chromosome 7, and *qPE22* and *qUI23* on chromosome 12, had consistent physical positions between PE and UI, implied that these loci could control both PE and UI.

The cloning of the key genes of PE and UI might be helpful to characterize the molecular mechanism. Hence, four novel loci (*qPE16*, *qPE21*, *qUI1*, and *qUI18*) were further explored to find causal genes for rice breeding. Through gene ontology analysis and functional classification, six potential functional candidate genes (*LOC_Os01g41420*, *LOC_Os07g38530*, *LOC_Os07g38810*, *LOC_Os08g15170*, *LOC_Os08g15280*, and *LOC_Os12g07690*) were ultimately identified, which were primarily involved in amino acid transport, energy metabolism, and protein kinases, suggesting they might be involved in regulating PE and UI in rice. However, whether they have a causal link to these traits remains to be further validated in transgenic plants in the future.

The *OsLIS-L1* (Gao et al. 2012) gene within *qPE19* was reported to regulate cell proliferation in UI, and the *CYP734A4* gene within *qPE14* and *qUI14* were reported to cause dwarfism in the mutant plants (Qian et al. 2017). Plant dwarfism has a significant correlation with UI according to the reports by Guan et al. (2011) and Duan et al. (2012). In our study, we also found the similar results that PE and UI in either *oslis-l1* or *cyp734a4* mutants were significantly shorter than those of WT, suggesting that *OsLIS-L1* and *CYP734A4* genes may be the causal genes with important regulatory role in PE and UI.

Superior alleles play an important role in breeding. To identify superior alleles for rice MAS, the haplotypes of *CYP734A4* and *OsLIS-L1* genes were analysed on PE, UI, and 1000-GW traits in this study. We found that average values of PE, UI, and PE/UI associated with Hap.3 of *CYP734A4* and Hap.3 of *OsLIS-L1* were lower than other types in 2015 and 2016, respectively. The 1000-GW of Hap.3 of *CYP734A4* and Hap.3 of *OsLIS-L1* was also reduced, as were PE and UI. In other words, Hap.1, Hap.2, and Hap.4 of *CYP734A4* and Hap.1, Hap.2, and Hap.4 of *OsLIS-L1* both increased PE, UI and 1000-GW. Furthermore, we found that significant differences between Hap.3 and Hap. 1/2/4 of *CYP734A4* and *OsLIS-L1* were caused by the significant SNP-6.23703769, SNP-8.3664103, SNP-8.3665235, and SNP-8.3666556 in promoter areas. Hence, these SNPs could be exploited as molecular markers to screen the superior alleles of the *CYP734A4* and *OsLIS-L1* genes, reduce panicle enclose and improve yield for rice breeding.

## Methods

### Plant materials

The natural population used for GWAS was composed of 205 rice (*Oryza sativa* L.) accessions from RDP1 inputted by Dr. Susan McCouch at Cornell University (Zhao et al. 2011). These accessions were divided into the *JAPONICA* group, which included 52 *tropical japonica* (*TRJ*), 59 *temperate japonica* (*TEJ*) and 3 *AROMATIC* accessions, and the *INDICA* group, which included 24 *AUS*, 33 *indica* (*IND*), and 34 *ADMIX* accessions. Detailed information regarding accessions is shown in Additional file 5: Table S2. Seeds were obtained by Dr. Jian Hua at Cornell University from the McCouch lab. The T-DNA mutants of the *CYP734A4* gene were provided by Dr. Wenzhen Liu (Qian et al. 2017), and the T-DNA insertion site and homozygous lines of *cyp734a4* were identified by PCR analysis of genomic DNA using gene-specific primers (Additional file 5: Table S6). The CRISPR/Cas9 mutants of *OsLIS-L1* were generated using the CRISPR/Cas9 system in this study.

### Field experiments

All accessions were grown in a paddy field at the Experimental Station of Nanjing Agricultural University (Jiangsu Province, China; 32.020° N, 118.500° E) in 2015 and 2016. Fifty plants of each accession were planted in one plot with a space of 17 cm between plants and 33 cm between rows, and each plot contained five rows (10 plants/row). For investigating of PE and UI traits, five plants in the middle two rows of each accession were randomly selected and were harvested at 35 days after heading. Their grains after 40 °C drying for 3 days were used for 1000-GW measurement.

### Generation of transgenic plants

The CRISPR/Cas9 plasmid was designed according to the protocol described previously (Xing et al. 2014). Two target sites of *OsLIS-L1* were confirmed via CRISPR-PLANT (http://www.genome.arizona.edu/crispr/CRISPRsearch.html). The target segment, totalling 964 bp, was obtained by amplifying the pCBC-MT1T2 vector using the BsF, F0, R0, and BsR primers, cloned into the pBUE411 vector, and then verified using the FD2, RD, and FD3 primers to develop transgenic plants. Genomic DNA was extracted from mutant seedlings using the cetyltrimethylammonium bromide (CTAB) method (Murray and Thompson, 1980). The PCR products were amplified with specific F and R primers and directly sequenced to detect the mutants. The positive mutants, in which that the second target site had a deletion mutant on the third coding exon, were finally selected. All primers are listed in Additional file 5: Table S6.

### Phenotype assessment

The leading panicle of each plant were selected to measure the PE and UI using a ruler. PE (in cm) was defined as the length from the sheath of the flag leaf to panicle base. When the base of the panicle was under the sheath of the flag leaf, a negative value was assigned. UI (in cm) was measured from the uppermost node to panicle base. The averaged values were used for data analyses. PE/UI was defined as the ratio of PE to UI. when the flag leaf overlapped a part of the PE, the value of PE/UI was marked as 0 < PE/UI < 1; when the flag leaf enclosed the panicle partly or fully, the value of PE/UI was marked as PE/UI ≤ 0; and when the flag leaf was under the node of the UI, the value of PE/UI was marked as PE/UI ≥ 1. In addition, HD was defined as when 80% of the leading panicle emerged from the leaf sheath in each plot (Lu et al. 2011). 1000-GW was measured using an electronic balance (measured to the nearest 0.0001 g).

### Genome-wide association analyses

The 700 K rice SNP marker set was used for GWAS as described by McCouch et al. (2016). Only the SNPs with nucleotide variations with missing rates < 0.25 and minor allele frequency (MAF) > 0.05 were used for GWAS in *INDICA*, *JAPONICA* and *All* populations (Yano et al. 2016). The result was SNP genotype data with 386,251 biallelic markers selected. These filtering steps were performed using TAEELE software version 5.0 (Bradbury et al., 2007). A linear mixed-model GWAS was then carried out using the EMMAX suite in Linux (Kang et al. 2010). The significance threshold *P* < 1.0e-4 was used, as in previous studies (Zhao et al. 2011; McCouch et al. 2016). Manhattan (Figs. 2 and 3) and Quantile-Quantile plots (Additional file 4: Figure S4) of GWAS results were made using the package qqman in R (Rebolledo et al. 2015). Significant-SNP clusters, that had clear peak-like signals and physical regions of any two significant SNPs were less than 200 kb, were considered to be one associated locus (Lu et al. 2016). The corresponding locus regions were determined by the significant SNPs on both ends. Candidate genes were predicted according to the Rice Genome Annotation Project MSU7 database (http://rice.plantbiology.msu.edu), and were classified into five functional groups according to Yano et al. (2016) based on all the significant SNPs in their regions.

### Haplotype analyses

For the *CYP734A4* and *OsLIS-L1* genes, the haplotypes were classified based on all SNPs with an MAF > 0.05 within functional range of the 5′ flanking sequence of a gene (≤2 kb from the first ATG) and the CDS of the target gene (Butardo et al. 2016). The haplotypes containing at least 5 investigated accessions were used for comparative analysis (Wang et al. 2015). Fisher’s least significant difference (LSD) test was conducted to compare the differences in PE, UI, and 1000-GW (He et al. 2015).

### Gene ontology analysis

A total of 306 candidate genes from four QTLs, *qPE16*, *qPE21*, *qUI1*, and *qUI18,* were submitted to the ‘AgriGO’ ontology enrichment facility (http://bioinfo.cau.edu.cn/agriGO/analysis.php) (Additional file 5: Table S4), which was based on Fisher’s exact test and a Yekutieli multi-test adjustment, using a 5% false-positive detection threshold.

### Statistical analyses

Phenotype data and variance (ANOVA) were analysed by Excel 2016 software. Broad-sense heritability (*H*_*B*_^2^) was calculated using the following equation:

$$ {H}_B^2=\frac{\sigma_{\tau}^2}{\sigma_{\tau}^2+\frac{\sigma_{\tau \beta}^2}{b}+\frac{\sigma^2}{bn}} $$

where ${\sigma}_{\tau}^2$ is the genotypic variance, *σ*_*τβ*_^2^ is the genotype-by-environment interaction variance, *σ*^2^ is the experimental error variance, n is the number of replicates and b is the number of environments. Correlation coefficients (LSD, *P* < 0.05) of PE, UI, PE/UI, 1000-GW, and HD were computed using PROC CORR in SAS software.

## Supplementary information

**Additional file 1. Fig. S1.** A heat map depicting Pearson’s correlation coefficients among PE, UI, 1000-GW and HD in 2015 (lower triangle) and 2016 (upper triangle) for 205 accessions in the study. * and ** indicate significant correlations at the 0.05 and 0.01 levels, respectively.

**Additional file 2. Fig. S2.** Gene structure and haplotypes of *CYP734A4* (A) and *OsLIS-L1* (B) identified in the CDS or promoter region. Green boxes and solid lines represent exons and introns, respectively. -49 T SNP (A) and -2547 T SNP, −1415G SNP, and -94C SNP (B) are the most significantly negatively associated with PE and UI.

**Additional file 3. Fig. S3.** Ontology analysis of candidate genes from *qPE16*, *qPE21*, *qUI1*, and *qUI18*. Proportions are shown for enriched (P < 0.05) functional categories. Data are obtained from comparisons of numbers of genes in the experimental (Input) and reference sets at ‘AgriGO’ (http://bioinfo.cau.edu.cn/agriGO/analysis.php). The blue bar represents the target candidates selected genes, and the red bar represents the rice reference genome genes.

**Additional file 4. Fig. S4.** QQ plots for PE and UI observed versus expected -log_10_(*P*) in *All* (A), *JAPONICA* (B), and *INDICA* groups (C) in 2015 and 2016. The red dashed line in each plot represents an idealized case where the theoretical test statistic quartile matches the simulated test statistic quartile.

**Additional file 5: Table S1.** The descriptive statistics of PE and UI for the *All*, *JAPONICA*, and *INDICA* groups in 2015 and 2016. **Table S2.** Information about the 205 accessions used in this study and phenotypic data of PE and UI observed in 2015 and 2016. **Table S3.** Genome-wide significant association loci of PE and UI for the *All*, *JAPONICA*, and *INDICA* groups in 2015 and 2016. **Table S4.** All the candidate genes identified for key novel loci *qPE16*, *qPE21*, *qUI1*, and *qUI18* (http://rice.plantbiology.msu.edu). **Table S5.** Candidate genes screened as functional classification of genes for key novel loci *qPE16*, *qPE21*, *qUI1*, and *qUI18*. **Table S6.** Primer pairs used for gene cloning and PCR analyses in this study.

## Data Availability

The datasets supporting the conclusions of this article are provided within the article and its additional files.

## Abbreviations

1000-GW: 1000-grain weight
GWAS: Genome-wide association studies
HD: Heading date
MAS: Marker-assisted selection
PE: Panicle exsertion
QTLs: Quantitative trait loci
RDP1: Rice diversity panel
SNP: Single-nucleotide polymorphism
UI: Uppermost internode
