# Supplementary figures and images for: Genome-wide association analysis of panicle exsertion and uppermost internode in rice (Oryza sativa L.)
Source: Rice (N Y). 2019 Sep 18;12:72. doi: 10.1186/s12284-019-0330-x (PMC6751241; doi:10.1186/s12284-019-0330-x)

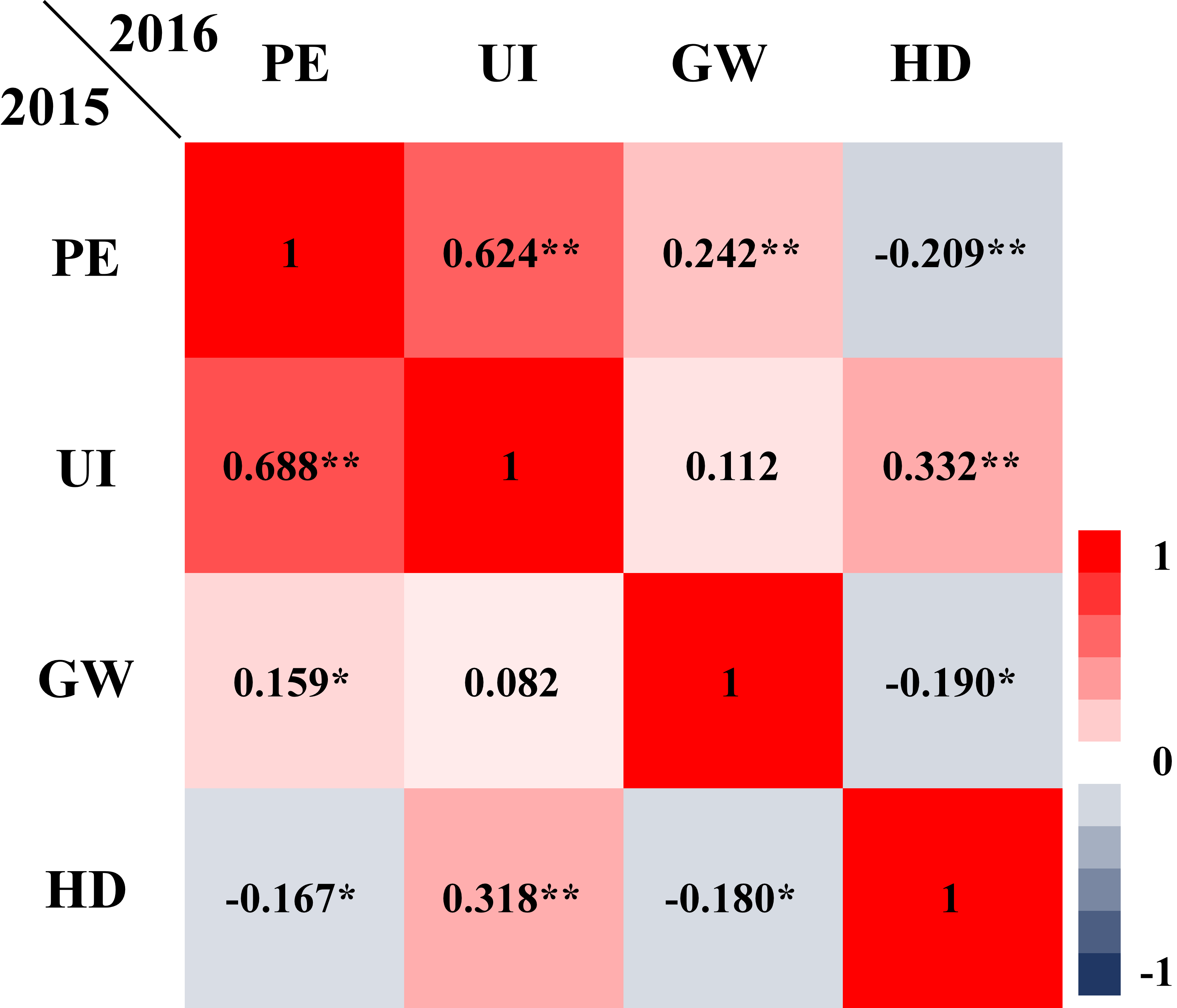

Supplement: Supplementary file 1 — Additional file 1. Fig. S1. A heat map depicting Pearson’s correlation coefficients among PE, UI, 1000-GW and HD in 2015 (lower triangle) and 2016 (upper triangle) for 205 accessions in the study. * and ** indicate significant correlations at the 0.05 and 0.01 levels, respectively. [file 12284_2019_330_MOESM1_ESM.tif]

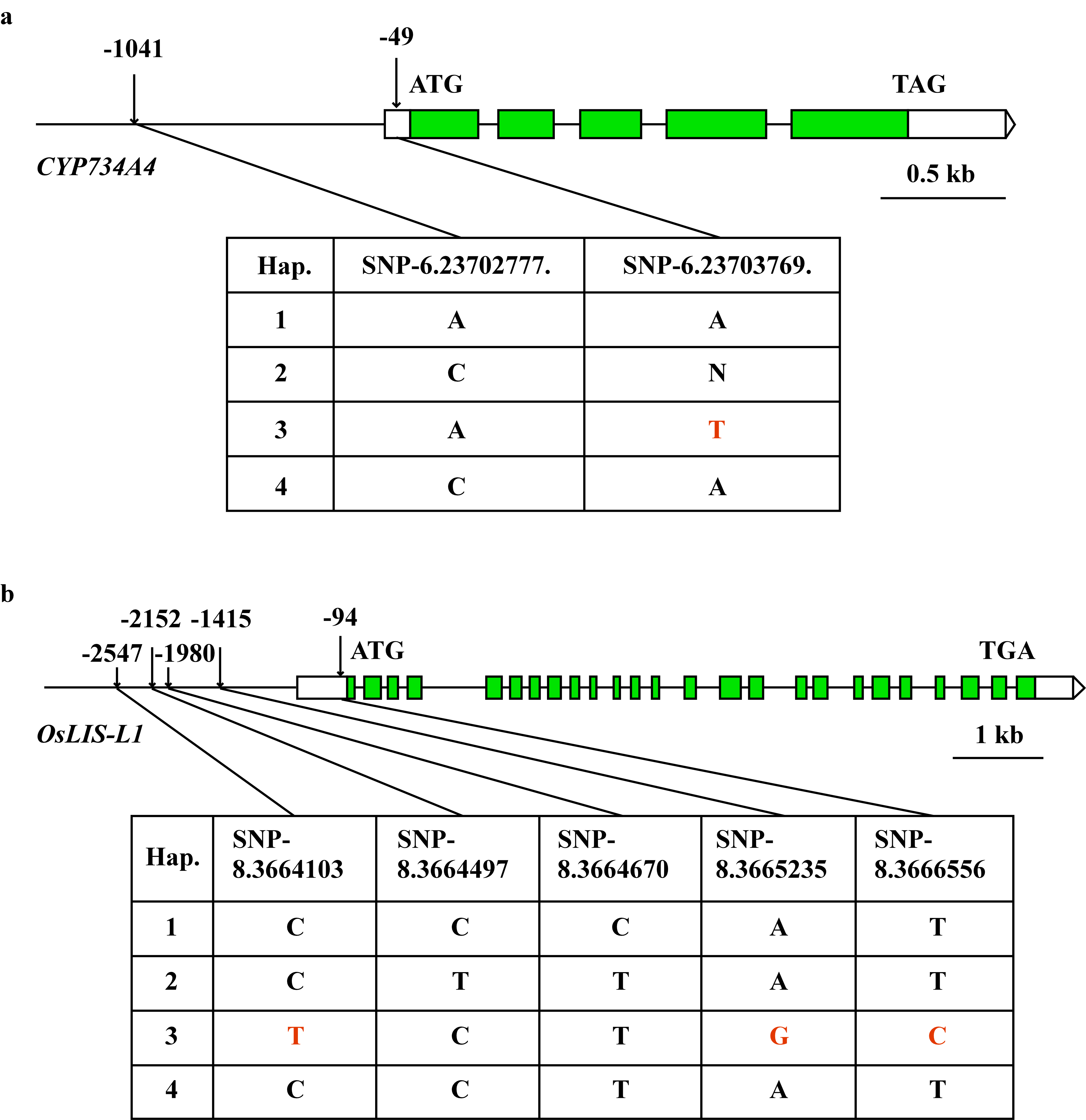

Supplement: Supplementary file 2 — Additional file 2. Fig. S2. Gene structure and haplotypes of CYP734A4 (A) and OsLIS-L1 (B) identified in the CDS or promoter region. Green boxes and solid lines represent exons and introns, respectively. -49 T SNP (A) and -2547 T SNP, −1415G SNP, and -94C SNP (B) are the most significantly negatively associated with PE and UI. [file 12284_2019_330_MOESM2_ESM.tif]

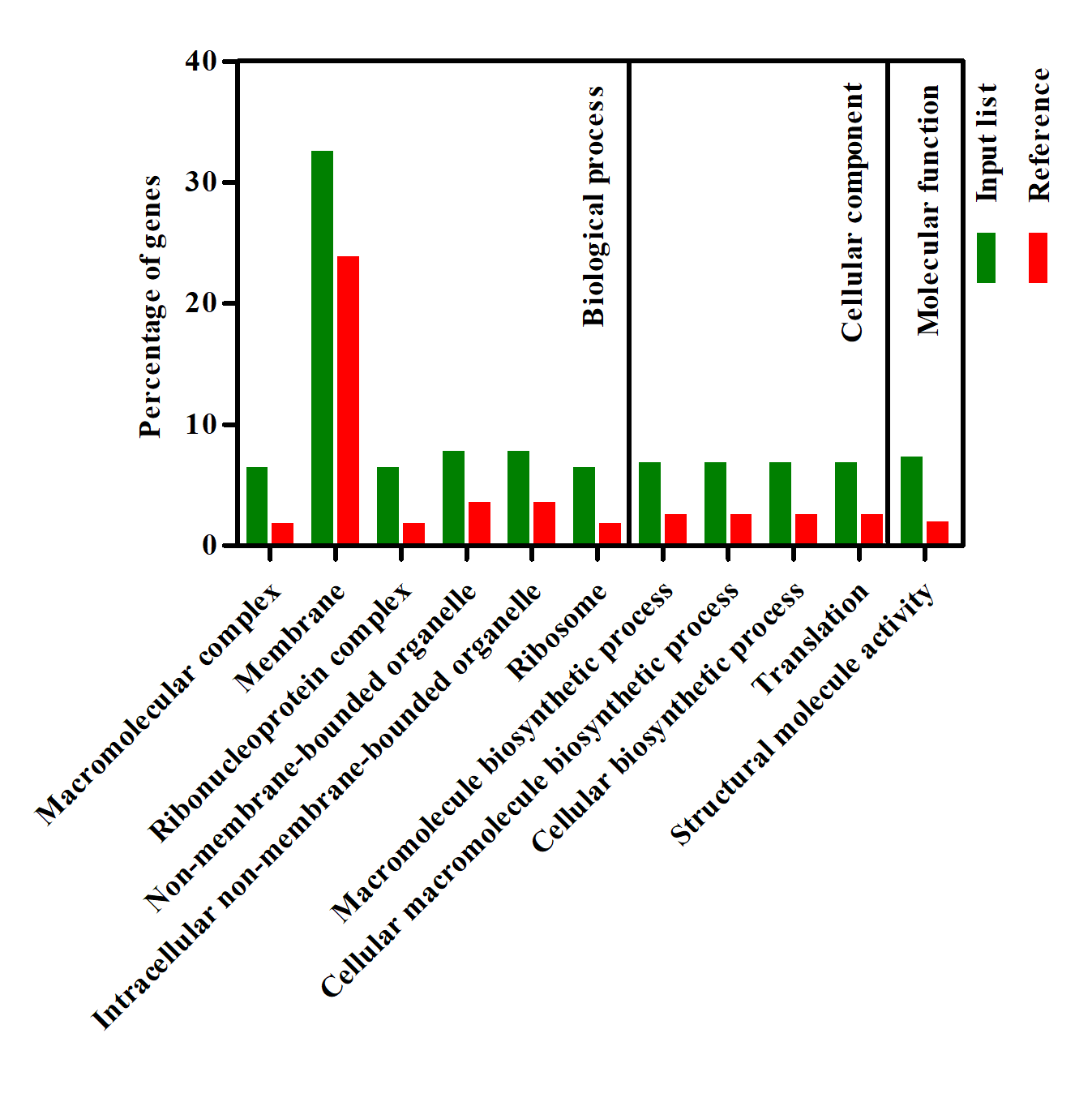

Supplement: Supplementary file 3 — Additional file 3. Fig. S3. Ontology analysis of candidate genes from qPE16, qPE21, qUI1, and qUI18. Proportions are shown for enriched (P < 0.05) functional categories. Data are obtained from comparisons of numbers of genes in the experimental (Input) and reference sets at ‘AgriGO’ (http://bioinfo.cau.edu.cn/agriGO/analysis.php). The blue bar represents the target candidates selected genes, and the red bar represents the rice reference genome genes. [file 12284_2019_330_MOESM3_ESM.tif]

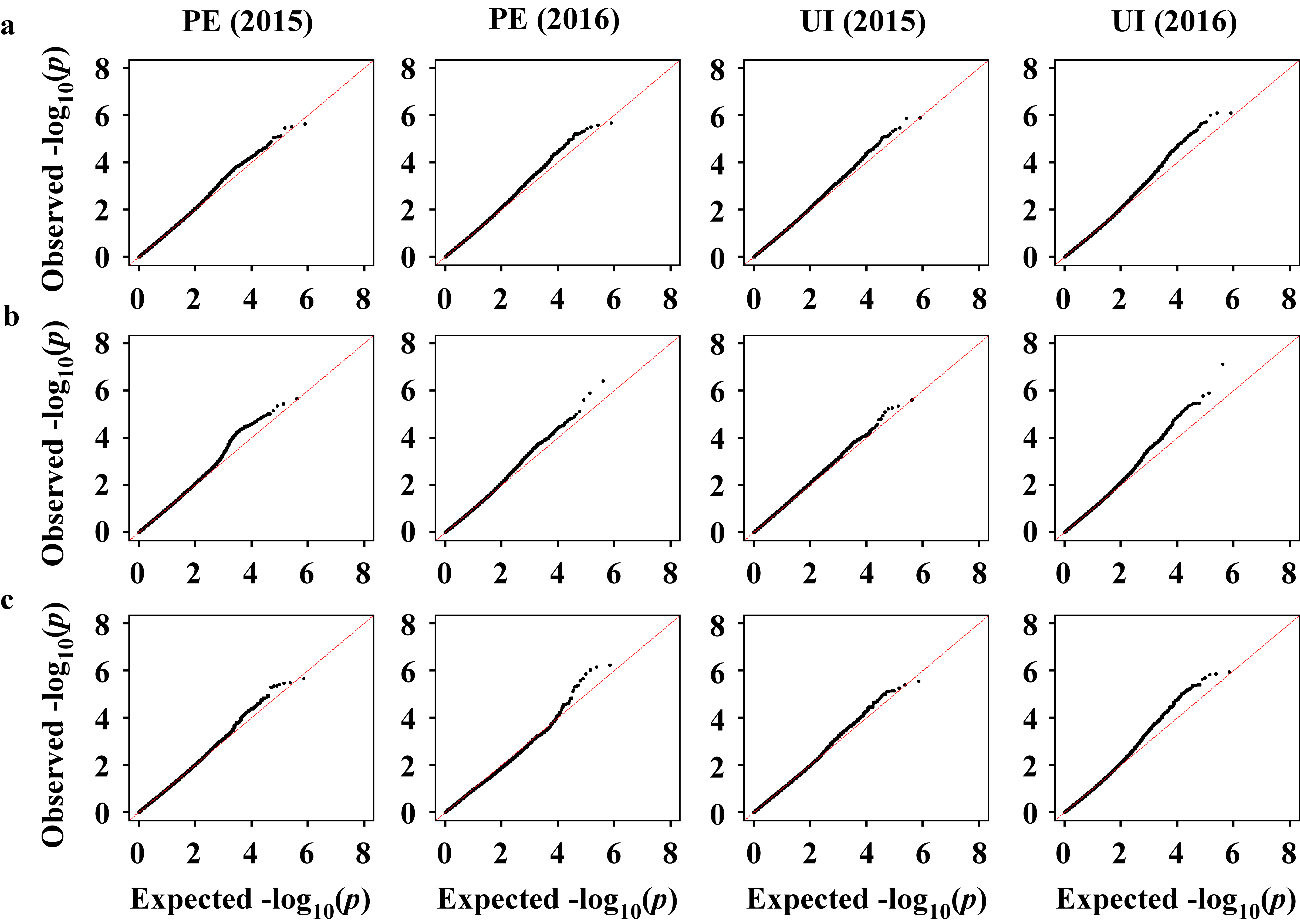

Supplement: Supplementary file 4 — Additional file 4. Fig. S4. QQ plots for PE and UI observed versus expected -log10(P) in All (A), JAPONICA (B), and INDICA groups (C) in 2015 and 2016. The red dashed line in each plot represents an idealized case where the theoretical test statistic quartile matches the simulated test statistic quartile. [file 12284_2019_330_MOESM4_ESM.tif]
